# Presyndromic surveillance for improved detection of emerging public health threats

**DOI:** 10.1126/sciadv.abm4920

**Published:** 2022-11-04

**Authors:** Mallory Nobles, Ramona Lall, Robert W. Mathes, Daniel B. Neill

**Affiliations:** ^1^ H.J. Heinz III College, Carnegie Mellon University, Pittsburgh, PA, USA.; ^2^ New York City Department of Health and Mental Hygiene, New York, NY, USA.; ^3^ Center for Urban Science and Progress, New York University, New York, NY, USA.

## Abstract

Existing public health surveillance systems that rely on predefined symptom categories, or syndromes, are effective at monitoring known illnesses, but there is a critical need for innovation in “presyndromic” surveillance that detects biothreats with rare or previously unseen symptomology. We introduce a data-driven, automated machine learning approach for presyndromic surveillance that learns newly emerging syndromes from free-text emergency department chief complaints, identifies localized case clusters among subpopulations, and incorporates practitioner feedback to automatically distinguish between relevant and irrelevant clusters, thus providing personalized, actionable decision support. Blinded evaluations by New York City’s Department of Health and Mental Hygiene demonstrate that our approach identifies more events of public health interest and achieves a lower false-positive rate compared to a state-of-the-art baseline.

## Teaser

Machine learning methods enable a new type of public health surveillance that acts as a “safety net” for public health.

## INTRODUCTION

To offer a rapid, targeted, and effective response to emerging biothreats, public health officials must be able to detect a huge variety of emerging events. Recent, high-profile events highlight the diversity of situations that can affect public health: In February 2020, 50+ residents of a nursing home in Kirkland, Washington were part of one of the first coronavirus disease 2019 (COVID-19) outbreaks in the United States; in October 2019, 100+ people contracted Legionnaires’ disease and Pontiac fever from hot tub displays at the North Carolina state fair; and in March 2018, 90+ people presented to emergency departments in five states with unexplained bleeding that was eventually traced to the use of synthetic marijuana laced with brodifacoum, or rat poison.

While existing, widely used disease surveillance systems such as the Centers for Disease Control and Prevention’s National Syndromic Surveillance Program have proven to be effective in detecting outbreaks of known illnesses, or those with common symptomology (e.g., influenza-like illness), these systems are not optimized for early detection of rare events or novel (previously unseen) biothreats. Syndromic surveillance systems typically monitor emergency department (ED) chief complaint data: free-text symptom data reported by each ED patient, recorded by a triage nurse, and sent to local public health organizations from hospitals in their jurisdiction. Chief complaints are typically short free-text strings, for example, “headache and pain in rt arm” or “cough and nasal congestion × 2 days.” These data are mapped to syndromes, like respiratory, fever, and gastrointestinal illness, and from there, spatial cluster detection methods, such as spatial scan statistics (*1*), or simpler time series methods are used to identify syndromes that are currently occurring with a higher-than-expected frequency in some geographic area (*2*–*5*). Once they are found, supplemental syndromes that describe novel events or rare illnesses can be added to the system (*6*). While developing syndromes in advance for every imaginable event is impossible, waiting until cases are observed to manually develop a new syndrome can result in substantial detection delays. Moreover, syndromic surveillance can dilute the signal of a rare outbreak or novel biothreat, either by grouping rare cases with more common illnesses, or by splitting cases among many syndromes. In either case, the syndromic surveillance system may require a large increase in cases to recognize an anomalous cluster corresponding to a rare or novel event, making it difficult for public health to achieve timely detection and response.

Given these fundamental limitations of syndromic surveillance, when the International Society for Disease Surveillance tasked a team of epidemiologists, public health practitioners, and technical analysts with translating public health’s most critical use-case deficiencies into well-defined technical problems, they first called for advances in “presyndromic” surveillance, a new type of surveillance that does not rely on assigning cases to existing or predefined symptom categories (*7*). Most existing methods for presyndromic surveillance use a keyword-based approach that compares word counts in the most recent period to word counts during a historical baseline period. These methods can report any occurrences of new keywords that were not previously seen in the historical chief complaints and identify anomalous word frequencies in the most recent data using various statistical methods including likelihood ratio tests, Poisson test statistics, and Fisher’s exact hypothesis test (*8*–*10*). However, keyword-based methods are unable to detect meaningful word combinations, and they frequently flag misspellings, typos, nonstandard abbreviations, or other nonmeaningful words and thus suffer from a high false-positive rate, making them impractical for daily use (*11*).

To address the critical need for new, effective, and deployable methods for presyndromic surveillance, we worked with local and federal public health organizations to design, develop, and test multidimensional semantic scan (MUSES). MUSES offers three notable methodological advances:

• MUSES eliminates the need for predefined syndromes by learning syndrome categories, including those that characterize rare or novel health threats and occur over a small number of cases, directly from free-text ED data.

• MUSES identifies localized case clusters through multidimensional spatial scan statistics, enabling detection of emerging biothreats that may be isolated to a certain hospital or spatial region or to a certain demographic group of patients.

• MUSES uses a practitioner in the loop approach to incorporate user feedback, “zoom in” on relevant patterns, reduce false positives, and provide local users with actionable insights based on their own criteria for what is, and is not, relevant.

**Fig. 1.**
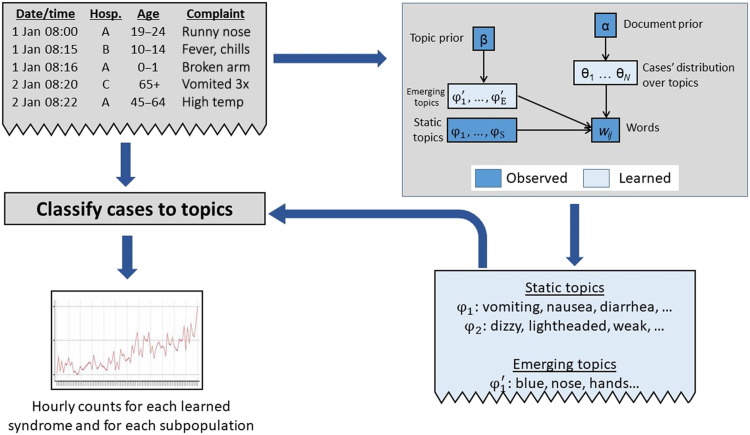
Overview of MUSES. MUSES is an innovative approach to presyndromic surveillance that learns newly emerging syndromes directly from free-text chief complaint data from hospital EDs, and detects statistically significant increases in cases related to these syndromes, including case clusters that may be limited to or differentially affect specific subpopulations.

An overview of MUSES is shown in Fig. 1, and we describe the approach in detail below. This study demonstrates that MUSES can serve as a “safety net” for public health surveillance by enabling the detection of emerging outbreaks and other events of interest that do not fit existing syndromes and might otherwise go undetected.

## RESULTS

### Learning syndrome categories directly from emergency department data

MUSES uses a new variant of topic modeling to learn syndrome categories directly from the data. Topic models are a set of algorithms that automatically summarize the main themes, or topics, contained in large collections of documents. The most widely used topic modeling approach, latent Dirichlet allocation (LDA), models topics as probability distributions over words and documents as probability distributions over these topics (*12*). Both the distribution over topics for each document and the distribution over words for each topic are learned automatically from a corpus of training data. In our setting, the documents are patients’ chief complaints and the learned topics act as syndrome categories, since they summarize symptoms that often appear together. Because standard topic models are designed to learn themes that best summarize the corpus as a whole, these topics correspond to common health conditions that occur frequently in the training data. For example, in our ED data, we might identify one topic with words corresponding to gastrointestinal illness (“vomiting,” “nausea,” “diarrhea,” etc.) and one with words corresponding to respiratory illness (“cough,” “dyspnea,” “shortness,” and “breath”).

To detect patterns that may represent emerging biothreats, we developed an extension of LDA that learns two sets of topics. First, we learn a set of 25 “static” topics by fitting a topic model to a set of historical data using the standard LDA approach. Then, we learn a second set of 25 “emerging” topics over only the most recent data using a new contrastive topic model (*13*). The set of historical static topics are designed to capture common events. Identifying clusters of such syndromes is not the main goal of our system; rather, we learn these common syndrome types to be able to differentiate them from newly emerging threats. The contrastive LDA model treats the historical static topics as observed parameters and optimizes the set of emerging topics to be maximally different from the historical topics. As a result, previously unseen words or words with new co-occurrence patterns dominate the set of emerging topics, which has the desired effect of capturing any new biothreats that occur in the most recent data. The contrastive LDA approach outperformed other extensions of LDA, including topics over time, online LDA, and labeled LDA (*14*–*17*), in detecting simulated disease outbreaks and identifying rare clusters in a variety of data settings (*13*).

### Detecting emerging anomalous clusters among subpopulations

After learning syndromes that can capture emerging biothreats, MUSES uses spatial scan statistics (*1*) to identify localized case clusters of these topics. Spatial scan has been used to identify emerging outbreaks of diseases including breast cancer, leukemia, and West Nile virus (*18*,*19*). Here, we search over all groups of ED cases defined by (i) one of the 25 learned emerging topics, (ii) a 1- to 3-hour time window of arrival to the ED, (iii) one hospital or all hospitals, (iv) a contiguous range of age groups, and (v) gender (males only, females only, or all). We note that the geographic and demographic information is not used when learning the topic models but only in the scan step. For each group, we compute a likelihood ratio statistic that describes the anomalousness of the observed number of cases relative to the corresponding expected baseline. Randomization testing is used to evaluate the significance of the highest-scoring group, adjusting for the multiple hypothesis testing issue, which could result from scanning over many subpopulations. This allows us to identify outbreaks of rare disease, or other novel biothreats, that may be isolated to a certain hospital and spatial region, or to a certain group of patients (e.g., the very young and very old).

### Detected relevant events in New York City

To evaluate the ability of MUSES to detect a diverse set of emerging patterns relevant to public health in large and complex data, we applied our algorithm to historical chief complaint data from New York City (NYC). This dataset has more than 28 million ED cases from 53 NYC hospitals during 2010–2016. For each hospital, we have data on the patients’ free-text chief complaint, date and time of arrival, age group, gender, and discharge International Classification of Disease–9 (ICD-9) diagnosis code. Public health practitioners at NYC’s Department of Health and Mental Hygiene (DOHMH) performed a blinded evaluation of the top 500 highest-scoring clusters detected over the 6-year time period by our method and by a competing, state-of-the-art, keyword-based approach. For each of these clusters, the evaluators indicated if the cluster (i) represents a meaningful collection of cases and (ii) is, in their judgement, highly relevant to public health (i.e., potentially worthy of follow-up investigation). For example, clusters related to “bacterial meningitis” and “synthetic drug use” were rated as highly relevant, clusters related to “motor vehicle accidents” were rated as meaningful but not highly relevant, and clusters resulting from misspellings or common words (such as “left”) were rated as not meaningful. We note that the evaluation was blinded (cluster lists for the two methods were merged and shuffled) so that the public health practitioner was not aware which method reported a given cluster or where that cluster ranked on its top 500 list.

**Fig. 2.**
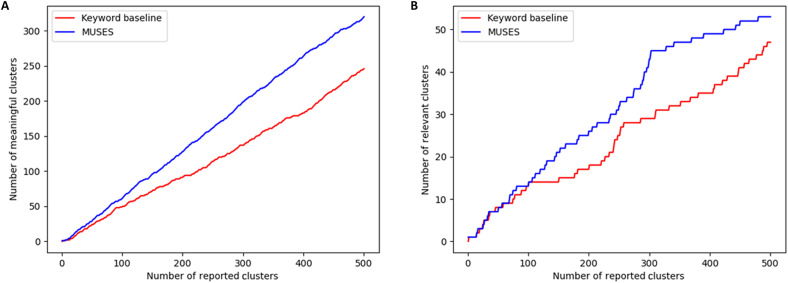
Results from a blinded user study comparing our MUSES approach (fixed model) to a competing, keyword-based approach. Each method’s top 500 highest-scoring clusters over a 6-year time period were rated as “meaningful and highly relevant,” “meaningful but not highly relevant,” or “not meaningful” by public health epidemiologists at NYC DOHMH. (**A**) Number of meaningful clusters and (**B**) number of “highly relevant” meaningful clusters, detected by each method, assuming that its top-*k* highest-scoring clusters were reported. Blue line: MUSES. Red line: keyword-based approach. For any fixed number of detected clusters, MUSES identifies more meaningful clusters and more highly relevant meaningful clusters than the keyword-based approach.

The blinded evaluation by DOHMH demonstrated that our method correctly identifies a larger number of events of interest to public health departments than the baseline method. We observe that 320 (64%) of the top 500 results from MUSES corresponded to meaningful health events, while the keyword-based method only detected 246 such events (49.2%). Figure 2A shows that for any fixed number of detected clusters, MUSES identified more meaningful events than keyword-based scan. Alternatively, for any desired number of discovered meaningful events, MUSES exhibits substantially higher precision: For example, to identify 100 meaningful events, it had to report 159 total clusters (precision = 63%) as compared to 225 total clusters (precision = 44%) for the keyword-based scan, corresponding to a 53% reduction in the number of false-positive clusters. Similarly, as shown in Fig. 2B, when 200 clusters are reported, MUSES detected 26 highly relevant clusters, while the keyword-based method detected 17. These findings demonstrate that regardless of the false-positive rate public health officials are willing to tolerate, MUSES offers an improvement over the current state of the art in presyndromic surveillance. This ability to report newly emerging case clusters that do not correspond to existing syndrome groups but have meaning and relevance to public health, without overwhelming the user with a large number of false-positive detections, suggests high potential utility for day-to-day operational use.

In addition, to determine how our approach might provide situational awareness of emerging health concerns following a natural disaster, we examined the clusters identified by our approach in the week following 29 October 2012, when Hurricane Sandy struck NYC and caused a historic level of damage. These results show a temporal progression of detected clusters from acute cases related to falls and shortness of breath, to mental health issues like depression and anxiety, to chronic health issues that require maintenance procedures, like dialysis and methadone distribution. Such procedures are typically handled in outpatient clinics but were displaced to EDs when clinics were closed because of storm damage and power outages. We note that DOHMH epidemiologists manually inspected hospital ED data immediately following Hurricane Sandy; noticed an increase in the words “methadone,” “dialysis,” and “oxygen”; and created a “needs medication” syndrome (*6*). The ability of MUSES to automatically identify similar symptoms as human experts highlights its ability to learn meaningful but previously unseen combinations of symptoms, including automatically identifying the progression of stresses on hospital EDs in the aftermath of a natural disaster.

### Incorporating practitioner feedback

While our initial, blinded evaluation with NYC public health officials confirmed MUSES’ ability to detect clusters of interest to public health, it also highlighted the potential benefits of developing a practitioner in the loop (PITL) approach. During the initial evaluation, DOHMH practitioners indicated that 102 of MUSES’ 200 highest-scoring clusters represented events that were meaningful collections of cases but not specifically relevant to their jurisdiction’s needs for the system, including 28 clusters related to motor vehicle accidents, 12 related to medical evaluations or clearances, and 8 related to alcohol intoxication. With a PITL approach, the public health user could mark the first identified occurrence of a “motor vehicle accident” as irrelevant to that particular health department, enabling the system to ignore or de-emphasize future instances of such clusters and to focus attention on known and relevant event types, as well as those that correspond to novel, previously unseen events. Human in the loop topic modeling has also been shown to increase topics’ interpretability, improve users’ ability to find information in a large corpus, and encode expert knowledge (*20*–*25*).

**Fig. 3.**
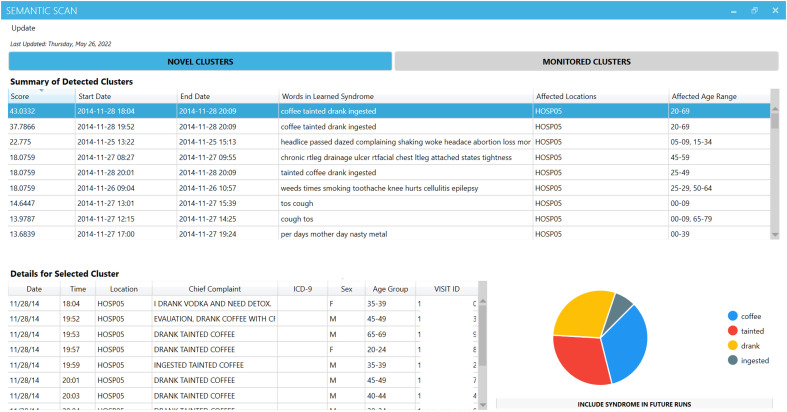
Screenshot of MUSES’ visualization interface after a cluster of cases related to drinking tainted coffee was detected in the data. This cluster is the highest-scoring cluster; thus, it appears first in the upper table. Because the user has clicked on this cluster, details about the cluster appear in the lower table and graph. The lower table shows de-identified clinical data associated with the nine cases in this cluster (the “Visit ID” column has been intentionally obscured), and the pie chart visualizes the learned syndrome’s distribution over words.

To capitalize on the benefits of including a human in the loop, we refined our system to efficiently collect feedback from public health practitioners about which alerts correspond to events of interest. Figure 3 shows that as public health practitioners use MUSES, they see a ranked set of detected clusters, each consisting of a list of ED cases and summary information about the spatiotemporal extent and textual topic of each cluster. The system allows public health officials to provide feedback on relevant events based on their own criteria and to distinguish between events of high interest (e.g., meningitis exposures) and low interest (e.g., motor vehicle accidents). This approach reduces the false-positive rate and allows public health officials to define events of interest based on their surveillance needs, without compromising the model’s ability to learn new syndrome types.

To incorporate practitioner feedback on a continuous basis into our contrastive topic model, we add two new classes of topics: monitored static topics and ignored static topics. As before, the model includes historical static topics to capture common events and emerging topics to capture rare diseases or novel biothreats. In addition, the model includes monitored static topics and ignored static topics, which were learned by a previous iteration of the model and marked by public health practitioners as events of high or low interest, respectively. The historical, monitored, and ignored static topics are all treated as known parameters by the contrastive topic model, and the scan step searches over both the emerging topics and the monitored static topics.

To measure the impact of the PITL approach, NYC public health officials participated in a second blinded experiment where they evaluated clusters detected by two versions of MUSES: one in which the model was iteratively updated with user feedback regarding clusters to monitor or ignore in the future and one in which the model remained fixed throughout the experiment. For each day in the experiment, the practitioner indicated whether the five highest-scoring clusters detected by each model represented collections of cases “to monitor,” “to ignore”, or that were “meaningless.” Every 2 weeks, the PITL model used this feedback to update its collection of monitored and ignored topics. DOHMH practitioners completed this experimental procedure over 19 two-week periods, corresponding to 9 months of historical data from January through September 2016, and 3.5 million data records from 53 hospitals.

**Fig. 4.**
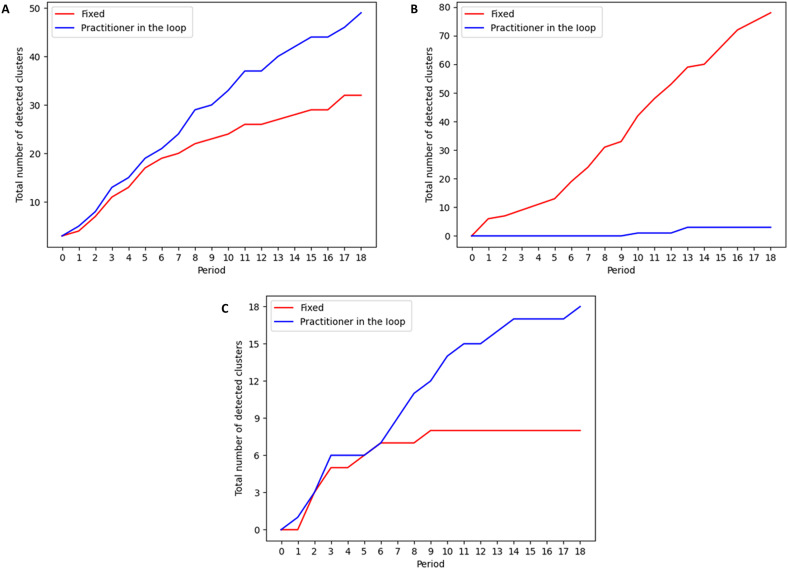
Results from a blinded user study comparing the fixed and PITL models. Blue lines: PITL model. Red lines: fixed model. (**A**) Cumulative number of highly relevant clusters detected by each method, after each 2-week time period. The performance gap between the PITL and fixed models increases monotonically as a function of the number of labeled clusters used as training data by the PITL model. (**B**) Cumulative number of clusters detected by each method that were similar to clusters previously labeled “to ignore” by the user, after each 2-week time period. During the experiment, the fixed model detected 78 irrelevant clusters similar to those labeled “to ignore,” while the PITL model only identified three such clusters. (**C**) Cumulative number of clusters detected by each method that were similar to clusters previously labeled “to monitor” by the user, after each 2-week time period. The PITL model identified a total of 18 highly relevant clusters that the practitioner had previously expressed interest in monitoring, as compared to 8 for the fixed model.

Evidence from this experiment supports four primary hypotheses: (i) The PITL model outperforms the fixed model with respect to precision and the number of detected events of interest; (ii) the performance gap between the PITL and fixed models increases monotonically as a function of the number of labeled clusters used as training data by the PITL model; (iii) after an initial labeling of a cluster as a topic to be ignored, the PITL model will avoid presenting similar clusters to the user in the future, thus reducing false positives as compared to the fixed model; and (iv) after an initial labeling of a cluster as a relevant topic to be monitored, the PITL model will have higher power to detect future instances of that topic. During the 19 periods included in the experiment, the PITL model detected 49 highly relevant clusters, a 53% increase over the 32 detected by the fixed model. Figure 4A shows that the PITL model had identified more events of interest during all periods of the experiment, but in period 3, the PITL model had detected 18.1% more relevant clusters (13 versus 11), whereas in period 10, this percent increase was 37.5%, and in periods 14 to 18, this percent increase was greater than 50% and significant at the 95% confidence level. To better understand these results, we consider our third and fourth hypotheses, which predict that the PITL model will present users with fewer clusters similar to those that the practitioner has deemed irrelevant, and more clusters similar to those in which they have expressed interest. Figure 4B shows that the fixed model detected 78 total clusters similar to those labeled “to ignore,” while the PITL model only identified three such clusters (a 96.2% decrease). The difference between the numbers of these clusters identified by the two models is significant at the 95% level for periods 1 to 18. Moreover, Fig. 4C shows that the PITL model detected 18 clusters similar to those labeled “to monitor,” while the fixed model only identified 8 (a 125.0% increase), and the difference between the numbers of monitored clusters detected by the two models is statistically significant in periods 14 to 18.

Because the fixed model could relearn an emerging topic that is similar to a monitored topic incorporated into the PITL model, if the cluster’s signal is sufficiently strong, then both models will detect the event. Nevertheless, as shown in Table 1, incorporating monitored static topics markedly improved the model’s ability to detect similar clusters in future iterations for five event types (falls, rash, cold weather exposure, gas exposure, and carbon monoxide exposure). In total, the PITL model detected 20 highly relevant clusters of these five types, as compared to 8 for the fixed model. In addition to detecting more examples of relevant clusters, Table 1 shows that the PITL model was also able to detect a greater variety of event types that public health users considered relevant, 24 event types as compared to 18 for the fixed model. Fifteen event types were detected by both fixed and PITL models, including those that may require emergency services (e.g., bathroom fire, pepper spray attack, and electrocution) and those related to potentially serious illness (e.g., meningitis, acute upper respiratory infection, and flu). The PITL model detected nine event types that the fixed model did not detect, while the fixed model only detected three events not identified by PITL. Furthermore, many event types only detected by the PITL model represent rare phenomena, like the cluster of four children and one adult who reported being exposed to lead paint in walls, or the cluster of six patients reporting “K2” (synthetic marijuana) drug use and anemia. On the other hand, events detected by only the fixed model include more common reasons for ED visits, like requests for medication or dialysis.

**Table 1.** Results from a blinded user study comparing the fixed and PITL models. For each of the 27 distinct event types identified at least once by users as highly relevant, the table compares the number of clusters of that type detected by the PITL and fixed models. The PITL model detected 49 highly relevant clusters corresponding to 24 distinct event types, while the fixed model detected 32 highly relevant clusters corresponding to 18 distinct event types. Clusters detected by the PITL model were further divided into those detected as “novel” clusters from emerging topics, and those detected from “monitored” static topics added by practitioner feedback. For five event types, incorporating monitored static topics improved the PITL model’s ability to detect similar clusters in future iterations.

**Event type**	**PITL model**					**Fixed model**
	**(Novel**	**+**	**Monitored**	**=**	**Total)**	
Falls	2	+	3	=	5	1
Rash	1	+	1	=	2	0
Cold weather exposure	2	+	1	=	3	1
Gas exposure	1	+	1	=	2	1
Carbon monoxide exposure	3	+	5	=	8	5
Smoke inhalation	1	+	7	=	8	9
Toxic inhalation/ fumes	2	+	0	=	2	0
Meningitis exposure	3	+	0	=	3	3
Pepper spray attack	1	+	0	=	1	1
Flu-like symptoms	1	+	0	=	1	1
Electrocution	1	+	0	=	1	1
Acute upper respiratory infection	1	+	0	=	1	1
Child medication ingestion	1	+	0	=	1	1
Syncope during marathon	1	+	0	=	1	1
Syncope among employees, possible meningitis	1	+	0	=	1	1
Bathroom fire	1	+	0	=	1	1
Substance abuse	1	+	0	=	1	1
Chemical burn	1	+	0	=	1	0
Gastroenteritis	1	+	0	=	1	0
Intoxication	1	+	0	=	1	0
Respiratory distress	1	+	0	=	1	0
Lead-based paint exposure	1	+	0	=	1	0
Drug use anemia	1	+	0	=	1	0
Gunshot wound	1	+	0	=	1	0
Chemical exposure	0	+	0	=	0	1
Needs dialysis	0	+	0	=	0	1
Medication refill	0	+	0	=	0	1
Total	31	+	18	=	49	32

Thus, we observe that the PITL MUSES model, incorporating monitored and ignored topics learned from user feedback, has improved ability to detect novel events, reduced false-positive rate, and can present users with more examples of clusters in which they have previously expressed high interest. Because PITL iteratively adds ignored and monitored topics to the model, and the emerging topics are chosen to be maximally different from these, it is able to better distinguish novel events from the known syndromes and patterns in the data. Furthermore, if the words in a cluster of documents are well represented by an ignored topic, they have a high likelihood of being assigned to this existing topic. Since we do not scan over subsets that include the ignored static topics, these clusters are unlikely to be shown to users, which reduce the false-positive rate. Conversely, because any documents that are closely aligned to one of the monitored topics have a high likelihood of being assigned to this monitored topic, fewer cases will be required to detect and report these events.

### Novel coronavirus (COVID-19) outbreak in NYC

The first wave of the COVID-19 pandemic hit NYC in March and April 2020 with catastrophic public health impacts. The city’s first COVID case was confirmed on 1 March, and by 30 April, there were over 174,000 confirmed cases and nearly 15,000 deaths, approximately one-fourth of the entire U.S. death toll to that point (*26*). Given the critical and long-lasting impacts of the pandemic, we retrospectively obtained (from NYC DOHMH) and analyzed ED chief complaint data from the 53 NYC hospitals for that time period, running MUSES (fixed model) for each hour of data from 1 March through 30 June 2020, and reporting the highest-scoring clusters. Additional ED data from 1 January through 29 February 2020 were used to learn static topics and to compute baselines for the scan.

The U.S. Centers for Disease Control and Prevention list of common symptoms of COVID-19 currently includes “fever or chills, cough, shortness of breath or difficulty breathing, fatigue, muscle or body aches, headache, new loss of taste or smell, sore throat, congestion or runny nose, nausea or vomiting, and diarrhea” (*27*). We hypothesized that because most of these (less severe) COVID symptoms are similar to commonly occurring illnesses (influenza, the common cold, and gastrointestinal illness), many COVID cases would be mapped to static topics rather than forming their own novel emerging topics. Thus, we labeled all of the static topics as “to monitor,” enabling MUSES to report clusters corresponding to both static and emerging topics.

In Table 2, we show the top 33 highest-scoring clusters detected by MUSES (with 25 static and 25 emerging topics and scanning over both static and emerging topics) during the first wave of the COVID-19 pandemic in NYC, from 1 March through 30 June 2020. We observe that MUSES was able to detect numerous, large, high-scoring clusters corresponding to the pandemic: 29 of the 33 clusters were detected between 18 March and 5 April, all of which were most likely due to COVID. These clusters included a range of COVID symptoms, including cough, fever, sore throat, shortness of breath, difficulty breathing, pneumonia, hypoxemia, body aches, headaches, and diarrhea. Three of the four highest-scoring clusters corresponded to a single hospital, with over 100 likely patients with COVID (within a 10- to 12-hour period) each day from 27 to 29 March. Some clusters described “screening,” “testing,” or “exposure,” but only 10 of the 29 clusters explicitly used the terms “covid,” “19,” or “coronavirus.” Of the remaining four of the 33 total clusters, the first cluster (on 17 March) included 42 cases (over an 8-hour period) complaining of smoke inhalation and/or coughing. As this is very large for a smoke inhalation cluster, it is likely that some of these cough cases were due to COVID rather than smoke inhalation. A second cluster (on 27 April) also included “covid screening” for 19 patients (over a 5-hour period) with cough, fever, and shortness of breath. In May 2020, the number of active COVID cases in NYC began to decline. Only two total clusters, unrelated to COVID, were detected in May (13 patients over a 1-hour period complaining of smoke inhalation) and June (9 patients over a 1-hour period complaining of bilateral tinnitus), respectively.

**Table 2.** Results from MUSES runs on ED chief complaint data from NYC DOHMH during the first wave of the novel coronavirus (COVID-19) pandemic in NYC, 1 March through 30 June 2020. Highest-scoring clusters found with 25 static and 25 emerging topics, scanning over both static and emerging topics. For each cluster, we report the date, de-identified hospital ID, number of cases, cluster duration in hours, whether the cluster is COVID-related, the most common chief complaints, and the cluster’s log-likelihood ratio score. ICD-10 diagnosis codes were noted when used consistently to describe cases in the cluster (9 of 33 clusters). At least 30 of the 33 detected clusters were COVID-related. Thirty of 33 clusters occurred during the peak of the pandemic in NYC (17 March through 5 April), and 32 of 33 clusters corresponded to emerging topics rather than static topics.

**Date**	**Hosp ID**	**No. of cases**	**No. of hours**	**COVID**	**Description**	**Score**
27 March	31	164	12	Y	“Covid 19 exposure,” flu-like symptoms, testing, cough, sob	244
28 March	31	152	10	Y	Testing, exposure, cough, sore throat, syncope	178
25 March	19	43	6	Y	“Coronavirus” [ICD-10: B97.29], cough, fever, headache, sob	75
29 March	31	111	11	Y	Testing, exposure, cough, fever, diarrhea, pneumonia	69
1 April	40	26	3	Y	Influenza-like respiratory [ICD-10: J10.1]	69
17 March	7	42	8	?	Smoke inhalation [ICD-10: J70.5], cough	65
26 March	1	14	3	Y	“Covid”, cough, sore throat, body ache, measured O2	58
2 April	52	64	11	Y	screening for viral disease [ICD-10: Z11.59], cough, fever, sob	54
27 April	7	19	5	Y	“Covid 19 screening”, cough, fever, sob	53
24 March	4	30	6	Y	Respiratory, headache	53
20 March	4	17	3	Y	Respiratory, vomiting, diarrhea, headache	52
24 March	14	14	3	Y	“Covid”, “wants covid”, cough, fever	52
4 April	38	14	4	Y	“Covid 19”, “covis 19”	50
26 March	31	23	5	Y	“Covid 19 exposed”, testing, flu-like symptoms	50
24 March	19	43	8	Y	“Coronavirus” [ICD-10: B97.29], cough, fever, sore throat	49
23 March	17	26	3	Y	Cough, fever	42
14 May	39	13	1	N	Smoke inhalation	41
30 March	31	37	4	Y	“19 testing”, difficulty breathing	41
5 April	31	13	2	Y	Flu-like symptoms	40
30 March	51	31	3	Y	Cough, fever	40
26 March	46	19	2	Y	Flu-like symptoms	40
21 March	14	47	7	Y	Cough, fever, chills, body ache	39
23 March	9	23	4	Y	Cough, fever, sob, face mask	39
24 March	14	25	3	Y	Cough, fever, headache, body ache	39
19 March	17	15	5	Y	Cough, fever, chills	39
31 March	40	28	7	Y	Influenza-like respiratory [ICD-10: J10.1] (monitored static topic)	39
1 April	32	11	4	Y	Flu-like symptoms	38
9 June	34	9	1	N	Bilateral tinnitus [ICD-10: H93.13]	38
1 April	52	22	4	Y	“Covid”, screening for viral disease [ICD-10: Z11.59], cough, fever	37
18 March	15	29	4	Y	Cough, fever, sickle cell crisis	37
20 March	19	15	3	Y	“Coronavirus” [ICD-10: B97.29], cough, fever	37
30 March	1	16	3	Y	Cough, fever, sob, pneumonia, lower resp. infection, hypoxemia	37
26 March	17	16	2	Y	Cough, fever, headache, body ache, diarrhea	37

Almost all of the detected clusters corresponded to the novel emerging topics as opposed to the monitored static topics. If we had only scanned over emerging topics, only one of the 33 clusters would have been missed: a cluster of 28 patients over a 7-hour period on March 31, complaining of influenza-like illness with respiratory manifestations. Thus, even without scanning over static topics, MUSES would have detected numerous case clusters resulting from the first wave of the COVID-19 pandemic in NYC. While one might expect clusters that explicitly mention “covid,” “19,” or “coronavirus” to be detected, since such terms were not present in the data used to learn static topics, it is interesting that commonly occurring terms like “cough,” “fever,” and “flu-like symptoms” were detected as “novel” clusters as well. For much of the dataset, cases containing these terms were indeed mapped to static rather than emerging topics, and we would expect this to reduce the detection power of MUSES for subtle, emerging outbreaks with common rather than novel symptomology. However, when a large cluster of cases emerges, as we observed in the COVID pandemic, MUSES learns emerging topics that are more precisely focused on common complaints for that cluster (e.g., cough and fever) and maps those cases to the emerging topics, enabling the cluster to be detected.

As a robustness check, we evaluated MUSES with three different numbers of static topics (10, 25, and 50) and compared the clusters detected (both for emerging topics and monitored static topics) in each case. The three variants produced extremely similar sets of detected clusters: The top 15 clusters for 10 and 25 static topics matched exactly, with minor differences in score (and thus some reordering of the cluster ranking), as well as minor differences in the age ranges included (and thus some differences in the precise set of cases included in each cluster). Fourteen of the top 17 clusters for 50 static topics also matched these 15 clusters, again with minor differences in score and cluster composition. While these results demonstrate that the number of static topics did not substantially affect MUSES’ ability to detect novel emerging clusters, some differences were observed in the clusters identified by scanning over monitored static topics. For 25 and 50 static topics, the same cluster of influenza-like respiratory illness on 31 March scored in the top 30 clusters, while for 10 static topics, no monitored static clusters scored even remotely close to the top 30. This difference was most likely due to the more focused topic distributions learned for larger numbers of static topics: Influenza-like respiratory illness formed its own topic for 25 and 50 static topics, while for 10 static topics, it was merged into a single topic with flu-like symptoms, cough, and fever. In addition, for 50 static topics, two other monitored clusters scored in the top 30: one cluster of 26 cases of flu-like symptoms in a 2-hour period on 27 March, and one cluster of 18 cough and fever cases in a 3-hour period on 21 March. Each of these two clusters was concurrent with a large, high-scoring novel cluster in the same hospital. While the assignment of COVID cases to static topics substantially reduced the score of the novel cluster for 1 hour of data, MUSES identified those cases as part of the larger novel cluster in the following hour, and thus neither timeliness nor accuracy of detection was significantly affected.

## DISCUSSION

While the above results demonstrate the potential utility of MUSES for identifying rare and novel events of public health interest, we now consider various limitations of the method that might reduce its ability to facilitate targeted and timely public health interventions. First, lags in data collection, preprocessing, analysis, or communication of results may affect timeliness, and thus effective use of MUSES depends both on a well-developed data infrastructure and the availability of public health practitioners to respond rapidly to the detected clusters. Second, false-positive clusters could result from repeated typographical errors by a particular triage nurse, hospital EHR changes and upgrades, or the use of new or unusual terminology to describe cases within a given hospital. While such clusters can easily be ignored, they may increase public health practitioners’ workload and potentially cause more relevant clusters to be overlooked. Thus, we have implemented data cleaning, including correction of common misspellings, as a preprocessing step for MUSES (as described below). Terminology changes could be incorporated either by adding them as ignored topics in the PITL model or by recomputing the static topics at regular intervals, as discussed below. Third, false negatives (i.e., failure to detect an emerging event of potential interest) could result from multiple sources of error. Chief among these are sampling bias and recording bias, since not everyone who has a particular symptom presents at the ED; those patients who do present may describe their symptoms differently, and different triage nurses may record them differently. ED usage may differ substantially between patient subpopulations depending on geographic, demographic, and socioeconomic characteristics, including factors such as access to care and insurance coverage. Given these biases, MUSES should not be used to perform population-level inferences like burden-of-disease estimation, nor should absence of a detected cluster be construed as an indicator that an event is not present. Rather, MUSES should be used as an exploratory data analysis tool, to identify potentially relevant case clusters that public health practitioners may otherwise have missed.

While MUSES does not assume or require a representative sample for detection, its statistical power to detect a given event will depend greatly on whether the affected subpopulation is overrepresented or underrepresented in the ED data, as well as the extent to which the resulting ED chief complaints use similar sets of words to describe each case. For example, if a newly emerging event is referred to using different terminology in different hospitals, then cases from different hospitals may be assigned to different emerging topics, potentially leading to both missed cases (since each topic’s case cluster will omit the cases assigned to other topics) and reduced detection power (since fewer cases will result in a lower score). Similar losses of power may occur due to typographical errors (though preprocessing will correct some of these) or inconsistent usage within a single hospital. However, if the distinct terms co-occur with each other (e.g., “headache – dolor de cabeza” in one hospital with a large Spanish-speaking population) or co-occur with the same other terms (e.g., “shortness of breahth” [*sic*]), they are likely to get grouped together into a single topic, mitigating this loss of detection power. In addition, an emerging pattern of cases may be grouped into an existing static topic rather than forming its own emerging topic, if it has too much overlap (in the sets of words used) with the existing topics. This incorrect grouping will lead to false negatives since the static topics are typically not included in the scan step. Possible solutions include using presyndromic surveillance as a complement to existing syndromic surveillance systems, which can pick up patterns of known syndromes, scanning over static as well as emerging topics, or using the PITL approach to designate certain static topics to be monitored rather than ignored. Last, our contrastive topic modeling approach is a randomized rather than deterministic algorithm, and thus MUSES is not guaranteed to identify identical clusters each time it is run on the same or similar data. However, we find in practice that the topics, and the resulting detected clusters, are highly consistent and robust to random variation when the amount of training data is large (e.g., for learning static topics) and when the signal is strong (e.g., the highest-scoring detected clusters are highly consistent across runs). For example, we ran MUSES on a 90% subsample of the original data from March to April 2020 and compared the top 30 clusters in the original data with the top 30 clusters in the subsampled data. We observed that 22 of 30 clusters in each list matched a cluster in the other list (with the same hospital, date, and time of day, and similar topics and cases), while the remaining clusters either narrowly missed the top 30 in the other list, or were narrowly beaten by a different cluster during that hour.

Given both the potential benefits and limitations of the method, we now consider how MUSES might be used operationally by a local health department, assuming both timely data availability and the availability of public health epidemiologists to examine and respond to the identified case clusters. Regular data feeds (e.g., daily or hourly) from hospital EDs to health departments are necessary for both syndromic and presyndromic surveillance and are already in place for many jurisdictions. For example, NYC DOHMH’s Syndromic Surveillance Unit in their Bureau of Communicable Disease collects data on a daily basis from all 53 NYC EDs and monitors case counts of common syndromes such as influenza-like illness, respiratory illness, and gastrointestinal illness. For presyndromic surveillance, the critical data fields for each hospital to collect and send to their local health department are the date, time, and free-text chief complaint for each ED case; additional data such as ICD codes, and demographics such as age and gender, can enhance both detection and follow-up investigation of clusters. An automated process could run MUSES daily on the most recent 24 hours of ED visit data, typically requiring no more than 20 to 30 min for the contrastive topic modeling and scanning steps and have results ready for practitioners to analyze each morning. Practitioners could peruse the top-scoring clusters, and ideally provide feedback, through the visualization interface each day, or only on days when the score exceeded some fixed threshold. This process would take no more than a few minutes, unless a cluster was deemed worthy of follow-up investigation. While monitored and ignored topics would be updated automatically from user feedback, it would also be desirable to update the static topics (requiring several hours of run time) at regular intervals, e.g., once every few months, to account for changes in case distribution or data entry practices. Last, we recommend using MUSES as a complement, rather than a substitute, to existing practices such as notifiable disease reporting and syndromic surveillance, as these existing approaches would be more effective for identifying patterns of known disease types and commonly occurring syndromes respectively. The relative portions of the public health workflow devoted to these tasks would be situationally dependent. For example, during the peak of the COVID pandemic, public health resources were almost entirely devoted to COVID response, while presyndromic surveillance could have been used in a more limited and focused way to identify newly emerging symptom patterns among patients with COVID.

MUSES builds upon new methodological approaches for syndrome discovery, cluster detection, and learning from user feedback to offer an innovative, presyndromic surveillance system that facilitates early detection and investigation of events of public health concern. Evaluation results from NYC DOHMH demonstrate the power of our detection methodology for accurately identifying clusters that are meaningful and relevant to local public health users, substantially improving the accuracy and specificity of detection as compared to existing state-of-the-art approaches. With the potential to enhance day-to-day situational awareness, to enable early detection of emerging biothreats during an emergency, and to provide a “safety net” to identify and investigate newly emerging and previously unseen events that existing systems would fail to detect, presyndromic surveillance is a critical next step for improved public health practice.

## MATERIALS AND METHODS

### Cleaning ED data

To clean the data, we first used the Emergency Medical Text Processor (EMT-P), an open-source, natural language preprocessing system (*28*). EMT-P standardizes chief complaint data by referring to the Unified Medical Language System, a thesaurus published by the U.S. National Library of Medicine to assist in linking terms in various electronic health systems. EMT-P was validated on 203,509 ED visits and an expert panel review of output found that the system’s corrections were 96% accurate (*28*). We also applied a spell checker trained on commonly occurring words in the chief complaint data and used a simple dynamic programming approach to infer if a word was missing a space (i.e., “COLDCOUGH”) and correct the error. Last, if an ICD-9 or ICD-10 code is included in the patient’s chief complaint field, we replace the numeric code with a textual description of the code provided by the Center for Medicare and Medicaid Services. Some NYC nurses include the emergency room diagnosis ICD code when recording chief complaints, and while these emergency room diagnosis ICD codes may differ from the final ICD codes assigned for billing purposes, they provide information about a patient’s observed symptoms.

### Learning syndrome categories

MUSES uses a contrastive topic model to learn syndromes that correspond to rare diseases or novel emerging biothreats. This extension of the LDA topic model learns two sets of topics: a set of *K*_S_ “static” topics over the historical data and a set of *K*_E_ “emerging” topics over only the most recent data. Each topic represents a probability distribution over words (i.e., a vector of length *V*, where *V* is the vocabulary size), learned from the data.

We learn the static topics by fitting a topic model to historical data using the standard LDA approach. To define a statistical model of the topics’ distributions over words and the documents’ distributions over topics, LDA makes a set of assumptions about how the data were generated. The standard generative model used in LDA assumes that each topic *k*’s distribution over words, φ*_k_*, and each document *d*’s distribution over the *K*_S_ static topics, θ*_d_*, are drawn from Dirichlet distributions with hyperparameters β and α, respectively. The Dirichlet is a distribution over the probability simplex and is the conjugate prior of the multinomial distribution. From there, each word *n* in each document *d* is assumed to be generated by first drawing a topic assignment *z*_*d*,*n*_ ~ multinomial(θ*_d_*) and then a word assignment *w*_*d*,*n*_ ~ multinomial(φ_*z*_*d*,*n*__) (*12*). These assumptions define the joint distribution *P*(φ, θ, *z*, *w*).

The topic-word distributions φ*_k_* and document-topic distributions θ*_d_* are latent (unobserved) and must be inferred from the data through the conditional distribution *P*(φ, θ, *z* ∣ *w*). While this posterior cannot be found through exact methods, there are a variety of methods that can be used to perform inference. MUSES uses collapsed Gibbs sampling, a common Markov Chain Monte Carlo approach, which is widely used for LDA models (*29*). This inference method starts by making random topic assignments *z*_*d*,*n*_ for each word *w*_*d*,*n*_, where *w*_*d*,*n*_ is the *n*th word in document *d*. Then, for every topic *k* and word *j*, we compute

$$\varphi_{k}^{\left(j\right)}=\frac{n_{k}^{\left(j\right)}+\beta }{n_{k}^{\left(.\right)}+V\beta }$$ (1)

where $n_{k}^{\left(j\right)}$ is the number of times word *j* is assigned to topic *k*, $n_{k}^{\left(.\right)}$ is the number of times any word is assigned to topic *k*, and *V* is the vocabulary size, or the number of distinct words in the corpus. Similarly, for every document *d* and topic *k*, we compute

$$\theta_{d}^{\left(k\right)}=\frac{n_{d}^{\left(k\right)}+\alpha }{n_{d}^{\left(.\right)}+K_{s}\alpha }$$ (2)

where $n_{d}^{\left(k\right)}$ is the number of times words in document *d* are assigned to topic *k*, $n_{d}^{\left(.\right)}$ is the number of times words in document *d* are assigned to any topic, and *K*_S_ is the total number of static topics. Following a common rule of thumb for LDA models, we use hyperparameters β = 1/*V* and α = 1/*K_S_*, where *V* is the vocabulary size and *K*_S_ is the number of static topics. Once we have these initial estimates of φ and θ, we loop through the words in each of the documents. In each iteration, we remove the topic assignment *z*_*d*,*n*_ of word *w*_*d*,*n*_, the *n*th word in document *d*. We update the estimates of φ and θ using Eqs. 1 and 2. Then, we estimate the likelihood that the word is assigned to each topic *k* by calculating

$$P(z_{d,n}=k)\sim \varphi_{k}^{w_{d,n}}\theta_{d}^{k}$$ (3)

We draw a sample from this distribution to get the new topic assignment *z*_*d*,*n*_ and re-update the estimates of φ and θ using Eqs. 1 and 2. This process continues until the topic-word and document-topic distributions converge.

### Learning rare syndrome categories

**Fig. 5.**
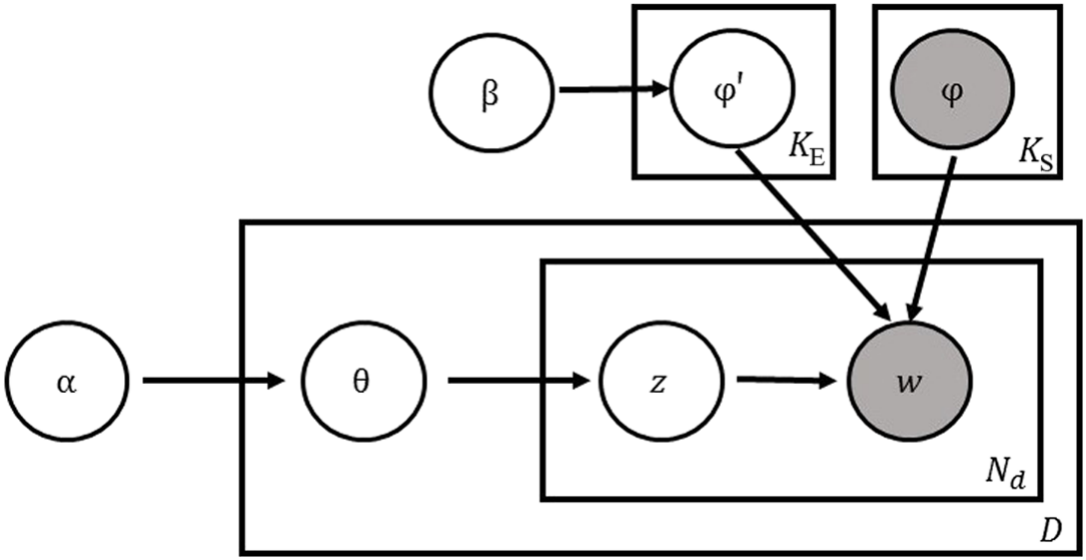
Plate diagram for the Emerging Topic LDA model.

To learn emerging topics that capture rare or novel biothreats, after we learn the set of static topics, we perform inference over our Emerging Topic Model, which is illustrated in Fig. 5 and makes the following generative model assumptions:

1) Each static topic φ*_k_* is a probability distribution over words (a vector of length *V*) that is observed and remains fixed at the levels learned by the static LDA model.

2) Each emerging topic $\varphi_{k}^{\prime }$is a probability distribution over words (a vector of length *V*) that is given by $\varphi_{k}^{\prime }\sim \text{Dirichlet}(\beta ).$

3) Each document *d* has a distribution θ*_d_* over static and emerging topics (a vector of length *K*_S_ + *K*_E_) that is given by θ*_d_* ~ Dirichlet(α).

4) For each word *w*_*d*,*n*_ in document *d*, a topic assignment *z*_*d*,*n*_ is drawn from Multinomial(θ*_d_*). If the topic assignment corresponds to a static topic, a word assignment is drawn from Multinomial(φ_*d*,*n*_). If the topic assignment corresponds to an emerging topic, a word assignment is drawn from $\text{Multinomial}(\varphi_{d,n}^{\prime })$.

Note that the presence of the fixed static topics distinguishes this model and set of assumptions from standard topic modeling. Given these generative assumptions, the joint distribution is

P(w,z,θ,φ′∣ φ,α,β)=p(z∣θ)p(θ∣α)p(φ′∣β)p(w∣z,φ′,φ) (4)

To learn the emerging topics’ distribution over words and the documents’ distributions over static and emerging topics, we perform Gibbs sampling with a few modifications. Here, we consider a total of *K* = *K*_S_ + *K*_E_ topics. Typically, we assume *K*_S_ = 25 static topics and *K*_E_ = 25 emerging topics. The initial topic assignments are based on the topics φ learned by performing inference on the historical corpus and the most recent collection of documents. That is, $P(z_{d,n}=k)\sim \varphi_{k}^{w_{d,n}}$, where *w*_*d*,*n*_ is the *n*th word in document *d*, *z*_*d*,*n*_ is the topic assignment of *w*_*d*,*n*_, and φ*_k_* is determined as follows: If *k* is a historical static topic, φ*_k_* was learned by applying standard LDA to a large set of historical data, and if *k* is an emerging topic, φ*_k_* was learned by applying standard LDA to the most recent set of data. After making the initial topic assignments, we proceed with the Gibbs sampling procedure described in the previous section, using the combined set of static and emerging topics, but only update φ*_k_* using Eq. 1 if *k* is an emerging topic, thus keeping all static topics fixed throughout the learning process. When this inference is complete, we refer to the second set of re-optimized φ′ as emerging topics.

We note that time is not explicitly represented in the Emerging Topic LDA Model (Fig. 5). Rather, the model can be used prospectively, running regularly (e.g., hourly) to detect emerging clusters. A moving window (typically 3 hours in length), stretching back from the current time, defines the “recent” data from which the emerging topics are learned and clusters are detected, thus producing a new set of detected clusters each hour.

### Incorporating practitioner feedback

It is straightforward to incorporate ignored and monitored static topics into the Emerging Topic Model. We assume that each ignored and monitored static topic *k* has a distribution over words $\varphi_{k}^{I}$ or $\varphi_{k}^{M}$ that is observed and remains fixed at the levels specified by the practitioner. These topics were learned by a previous iteration of the contrastive LDA model, and the practitioner may make manual changes to the ignored or monitored topic’s distribution over words before adding it to the model. To learn emerging topics, we use the modified Gibbs sampling procedure described above and treat ignored and monitored topics as static topics.

### Scoring the anomalousness of detected clusters

After learning newly emerging syndromes from the chief complaint data, we use a multidimensional extension of spatial scan statistics to identify localized case clusters corresponding to these topics. Kulldorff’s spatial scan approach considers circular geographic search regions that are centered at each monitored location and have varying radii, allowing for detection of both spatially compact and dispersed clusters. Extensions to this method consider a variety of types of geographic search regions, including rectangles, ellipses, and more general search regions over subsets of the data (*30*, *31*). In this study, each group of patients that we wish to consider can be represented by a temporal-spatial-demographic search group *S*

$$\begin{array}{c} S=\{\varphi^{\prime },\varphi^{M}\}\times (t_{\text{start}},t_{\text{end}})\times \{h_{1},h_{2},\ldots ,h_{n}\}\times \\ \left\{\delta_{1,1},\delta_{1,2},\ldots ,\delta_{1,p}\}\times \ldots \times \{\delta_{j,1},\delta_{j,2},\ldots ,\delta_{j,s}\right\} \end{array}$$

A patient is included in search group *S* if and only if the patient was seen at one of the hospitals in {*h*_1_, *h*_2_, …, *h_n_*} during the time window (*t*_start_, *t*_end_), has demographic characteristics included in {δ_1,1_, δ_1,2_, …, δ_1,*p*_} × … × {δ_*j*,1_, δ_*j*,2_, …, δ_*j*,*s*_}, and has a chief complaint that maps to a given emerging topic or monitored static topic (note that we do not scan over the historical or ignored static topics). To map each chief complaint to one of the learned syndromes, we follow (*13*), and first compute the probability of each word in the chief complaint being assigned to each topic, using the current values of φ and θ. Then, the values of θ are updated on the basis of the computed probabilities, and the process iterates. Once convergence has been reached, the chief complaint is assigned to the topic with the highest probability in θ.

For each search group, we compute the log-likelihood ratio score

$$F(S)=\text{log}\frac{\text{Pr}(\text{Data}\;|H_{1}(S))}{\text{Pr}(\text{Data}\;|H_{0})}$$ (5)

where the null hypothesis *H*_0_ is that there is not a cluster of any topic, and the alternative hypothesis *H*_1_(*S*) is that there is a cluster of some (emerging or monitored static) topic affecting search group *S*. We assume that if there is no cluster, count *c*_*i*,*j*,*k*_ of this topic at time *i*, location *j*, and among demographic group *k* will be distributed according to a Poisson distribution *c*_*i*,*j*,*k*_ ~ Poisson (*b*_*i*,*j*,*k*_), where *b*_*i*,*j*,*k*_ is a baseline or expected count for this topic during the same time frame and among the same demographic group of patients. If these patients are affected by a cluster, we expect that there will be a multiplicative increase in counts as compared to the baseline; i.e., the count will be distributed as *c*_*i*,*j*,*k*_ ~ Poisson (*qb*_*i*,*j*,*k*_) for some constant *q* > 1, where *q* is estimated by maximum likelihood. Given these assumptions, the formula for the log-likelihood ratio score *F*(*S*) simplifies to

$$F(S)=\{\begin{array}{c} C\;\text{log}\frac{C}{B}+B-C,\text{if}\;C>B\\ 0,\text{if}\;C\leq B \end{array}$$ (6)

Here, $C=\sum_{i,j,k}c_{i,j,k}$ and $B=\sum_{i,j,k}b_{i,j,k}$ represent aggregate case counts and estimated baselines for the given topic over the search group for the considered time period. Baselines (expected counts) account for variations in case count by time of day. The baseline for time period *i* is given by *b_i_* = (AC_h_ + AC_oh_)/2, where AC_h_ is the average hourly case count over the last 28 days during the same hour as the hour in time period *i*, and AC_oh_ is the average hourly case count over the last 28 days during all other hours of the day. We use *F*(*S*) to identify the highest-scoring clusters of cases that should be reported to users as potential events of interest.

We use the log-likelihood ratio scan statistics to identify anomalous clusters in both MUSES and the keyword-based comparison method. While keyword-based approaches have used a variety of statistical methods for this task, this allows us to isolate and study the impact of learning syndromes versus considering each keyword individually.

### Blinded user studies

For the first blinded user study, we ran both MUSES (fixed model) and the competing keyword-based approach for each hour of data over the entire 6-year period from 2010 to 2016. For a given hour of data, we used a 3-hour moving window (that hour and the two previous hours) to learn emerging topics and scanned over clusters from 1 to 3 hours in duration. The static model for the first case study was learned from a 10% sample of the entire 6 years of data. For this user study, we learned separate topic models for each NYC hospital, with 25 static and 25 emerging topics for most of the hospitals, and up to 50 static topics for hospitals with particularly large patient populations.

For the second blinded user study, we ran both the fixed and PITL models for each hour of data in 2-week increments from 1 January through 30 September 2016. For a given hour of data, we used a 3-hour moving window (that hour and the two previous hours) to learn emerging topics and scanned over clusters from 1 to 3 hours in duration. The initial static topic model (for both fixed and PITL models) was learned from the previous year of data, 1 January through 31 December 2015. For this user study, we learned a single topic model across all NYC hospitals, using 25 static and 25 emerging topics. The PITL model’s static topics were updated after each 2-week period (adding monitored and ignored static topics as labeled by the user), while the fixed model’s static topics were not updated.

### Evaluating the impact of the PITL

To understand why the PITL model offers improvements over the fixed model, we first consider our third hypothesis, which predicted that after an initial labeling of a cluster as a topic to be ignored, the PITL model will avoid presenting similar clusters to the user in the future, thus reducing false positives as compared to the fixed model. We evaluate this hypothesis by measuring the total number of clusters detected by the model in each period that have topics similar to a topic that was previously detected and labeled “to ignore” by the public health practitioner. That is, the number of previously incorporated ignored (PII) topics shown to the user in period *x* is given by

$$\text{PII}(x)=\sum_{i=1}^{x}\sum_{c\;\epsilon \;I_{i}}\sum_{t\;\epsilon \;{\text{IST}}_{i}}I(\text{similarity}(c,t)>\tau )$$ (7)

where *c* is a cluster, *I_i_* is the set of clusters detected by the model and labeled “to ignore” by the practitioner in period *i*, *t* is a topic, IST*_i_* is the set of ignored static topics at the start of period *i*, similarity is a similarity measure, and τ is a similarity threshold. Here, we measure the similarity between a detected cluster *c* (with a topic *t_c_*) and an ignored topic Tby evaluating

$$\text{similarity}(c,t)=\sum_{w}\text{min}(\varphi_{t_{c}}^{w},\varphi_{t}^{w})$$ (8)

where $\varphi_{t_{c}}^{w}$ and $\varphi_{t}^{w}$ are the probabilities of word *w* in the cluster’s topic and the ignored topic, respectively. For example, the similarity between a detected cluster with a topic that places probabilities of (0.4,0.4,0.2) on the words (motor, vehicle, crash) and an ignored topic that places probabilities of (0.33,0.33,0.34) on the words (motor, vehicle, accident) would be min(0.4,0.33) + min (0.4,0.33) + min (0.2,0) + min (0,0.34) = 0.66. A similarity threshold of τ = 0.6 was used for this analysis.

Our final hypothesis predicts that after an initial labeling of a cluster as a relevant topic to be monitored, the PITL model will have higher power to detect future instances of that topic. We evaluate whether the PITL model is able to identify more clusters that are similar to those the practitioner has labeled “to monitor” than the fixed model by measuring

$$\text{PMI}(x)=\sum_{i=1}^{x}\sum_{c\;\epsilon \;M_{i}}\sum_{t\;\epsilon \;{\text{MST}}_{i}}I(\text{similarity}(c,t)>\tau )$$ (9)

where *M_i_* is the set of clusters detected by the model and labeled “to monitor” by the practitioner in period *i*, MST*_i_* is the set of monitored static topics at the start of period *i*, and all other values are defined as in Eq. 8.
